# There’re CRISPRs in My Yogurt: A Discovery-Based CURE at the Intersection of Industrial Food Production and the Human Microbiome

**DOI:** 10.3389/fmicb.2020.578737

**Published:** 2020-10-22

**Authors:** Katherine L. Petrie

**Affiliations:** ^1^Division of Biological Sciences, University of California, San Diego, La Jolla, CA, United States; ^2^Earth-Life Science Institute, Tokyo Institute of Technology, Tokyo, Japan

**Keywords:** lactic acid bacteria, yogurt, gut microbiome, CRISPR, bacteriophage resistance, CURE, inquiry-based lab

## Abstract

Support for undergraduate laboratory education based on a CURE (Course-based Undergraduate Research Experience) model is more widespread than ever. By giving students the opportunity to conduct genuine research in laboratory courses they are required to take, CUREs can expose more students to scientific practice and have the potential to make science more inclusive, especially when research topics have direct impact on students’ lives. Here, I present a new microbiology CURE module where students explore the real-world intersection between industrial food production and the human microbiome. In this module, students sequence CRISPR arrays in the genomes of lactic acid bacteria they isolate from yogurt. Natural CRISPRs (Clustered Regularly Interspaced Short Palindromic Repeats) act as the bacterial immune system. When a bacterial cell survives viral infection, it can incorporate a bit of that virus’s DNA into its own genome, and produce small RNA guides that surveil the cell, ready to deploy virus-destroying enzymes if matching DNA from a fresh viral infection is detected. This viral immunity is of particular interest in the fermentation industry, since viral infection can destroy stocks of starter cultures and batches of product. Commercial producers of lactic acid bacteria for yogurt production often endeavor to produce strains with large CRISPR arrays and robust immunities. With this context, students are given the task of cataloging the viral immunities found in both commercial and traditionally produced yogurt, and exploring their potential impact on human health. Wet-lab practices (strain isolation, PCR, and Sanger sequencing) are combined with bioinformatic and literature sleuthing to identify the viruses to which bacteria are immune and explore whether consumption of these strains could impact human health via interactions with the human microbiome. Here, a detailed implementation of the module is presented with guides for educators and students.

## Introduction

### Educational Context

In the past decade or two there has a been an increasing call to make the science students learn in laboratory courses more reflective of the way science is actually conducted (American Association for the Advancement of Science [AAAS], 2011). One way to do this is through research projects that are open-ended and more authentic than standardized cookbook style activities. In these projects, not only do the students not know what the results might be, but neither does the instructor, nor the scientific community at large. Such projects, where the results have potential novel relevance to science, have been dubbed CURES — Course-based Undergraduate Research Experiences (Auchincloss et al., 2014). CURES can make science more inclusive by providing research opportunities for students who, due to economic hardship or other systemic barriers, are unable to obtain a traditional, apprenticed, extracurricular research position in a laboratory (Bangera and Brownell, 2017).

Because of these potential benefits, CUREs have become increasingly widespread. One of the most well-known CUREs, the nationwide SEA-PHAGES project, first got its start in the early 2000s (Hanauer et al., 2006). Since that time, countless CURES have been developed, both by individual instructors and as multi-campus initiatives. Along with the increased development and adoption of CUREs, a large body of work has been produced that studies their impact on students. These studies aim to identify which components of CUREs make them beneficial to students, and also provide a framework for how CUREs may benefit students by increasing persistence in science.

What has this work shown? In 2014, a working group from the NSF-funded Course-Based Undergraduate Research Experience Network (CUREnet) published an operational definition of a CURE, based on five components, or dimensions, of research (Auchincloss et al., 2014). According to the CUREnet report, authentic CURES that represent genuine research should embody all five dimensions (Table 1). How do these dimensions benefit students? Participation in course-based research has been shown to improve student understanding of the nature of science (Russell and Weaver, 2011) and promote scientific thinking as well as the ability to interpret data (Brownell et al., 2017). In addition to these cognitive gains, studies have also looked at the affective and psychosocial impacts of student participation in CUREs. Work has shown that project ownership, self-efficacy, science identity, and adoption of scientific community values can be influenced by CUREs (Hanauer et al., 2016; Cooper et al., 2019). Students who measure higher in these areas are more likely to persist in science, and this is especially true for students from underrepresented populations (Hanauer et al., 2016; Corwin et al., 2018). CURE researchers are still pursuing a full understanding of how individual CURE components and activities contribute to these outcomes, how much of each component in a course is sufficient to see gains, and how different student populations may see different impacts (Auchincloss et al., 2014).

**TABLE 1:** The five dimensions of research in CUREs and how they are represented in this module.

	**Dimension of research**	**Representation in “There’re CRISPRs in my yogurt” module**
1	Multiple scientific practices (students do more than just collect data)	Students collect data according to project’s scientific goals, but they also analyze data, and in lab reports, interpret data, communicate their findings, and propose future experiments.
2	Discovery (results are unknown to students and instructors)	CRISPR arrays of commercial and heirloom yogurt bacterial strains have not been compared, and it is unknown if and how consuming strains with altered viral immunities may affect the gut microbiome.
3	Relevance (there is potential for broad relevance beyond the scope of the course)	Food microbiology and the human gut microbiome are fields with broad general interest; there are many unanswered questions in our understanding of interactions between diet and the gut microbiome.
4	Collaboration	Students collaborate on multiple levels: they coordinate with their partner and group to carry out experiments, and use shared data from the entire class to draw conclusions.
5	Iteration	Students carry out repeated screens of multiple strains, and the module builds in time to repeat experiments if there are failures. There is also iteration from course to course, as lessons are learned and new strains are isolated and analyzed.

*Dimensions adapted from CUREnet working group report (Auchincloss et al., 2014).*

This paper presents a CURE module, “There’re CRISPRs in my Yogurt” that could be incorporated into an undergraduate microbiology laboratory course. The module would be most appropriate for junior or senior students.

### Scientific Context

The goals of this and most CUREs are two-fold. The first goal is to give students exposure to genuine research and thereby give them a better understanding of how new knowledge is constructed. The second goal is to contribute to scientific understanding. Here, the CURE explores CRISPR-based viral immunity in lactic acid bacteria used to produce yogurt for consumption. The ultimate goal is to create a catalog of viral immunity in food strains, which can be used as a reference to better understand potential interactions between the gut microbiome and the foods we eat.

While many students have heard of CRISPR in the context of its use in genetic engineering, they are less aware of the function of natural CRISPRs. Researchers first observed the pattern of CRISPRs (Clustered Regularly Interspaced Short Palindromic Repeats) in *E. coli* (Ishino et al., 1987). In a given bacteria, they consist of arrays of conserved repeat sequences of ∼21–40 bp, separated by non-conserved spacer sequences of ∼20–58 bp (Ishino et al., 2018). The key moment in discerning their function came when researchers noticed that the spacer sequences matched foreign DNA sources — bacteriophage, prophages, and phagemids — and that bacteria which harbored spacers were resistant to infection by the corresponding virus (Mojica et al., 2005; Pourcel et al., 2005). This immunologically protective function of CRISPRs was experimentally confirmed in *Streptococcus thermophilus*, by researchers affiliated with Danisco, a company producing bacterial starters for the food industry (Barrangou et al., 2007; Lander, 2016).

The molecular details of how cas proteins incorporate bits of viral DNA into the host CRISPR array and then use guide RNAs derived from them to target foreign DNA in subsequent infections were figured out over the next decade (Lander, 2016; Ishino et al., 2018). Phage-resistant bacteria are of interest to companies that produce lactic acid bacteria strains for the dairy industry, since phage infection can ruin industrial cultures (Lander, 2016). Since the discovery of their function, commercial companies have systematically created phage-resistant bacterial strains by a process akin to vaccination: bacteria are exposed to phage, and surviving bacteria are screened for the presence of CRISPRs (Grens, 2014; Barrangou and Horvath, 2017).

Because of their different natural histories, and because some commercial strains may have been intentionally vaccinated, the CRISPR loci of industrially produced commercial yogurt bacteria may differ, in both size and spacer identity, from the CRISPR loci of traditionally produced heirloom yogurt. Modern commercial yogurt is produced by fermentation of milk with a few well-defined isogenic strains. Here, heirloom yogurt refers to yogurt made from a mother culture containing an undefined mixture of wild strains (Fisberg and Machado, 2015). Yogurt is generally lauded for its broad health benefits (Fisberg and Machado, 2015), and its potential to positively influence the human gut microbiome (Veiga et al., 2015), but interactions of yogurt bacteria with the commensal microbiome are not fully characterized.

The human gut microbiome includes a bacteriophage component which varies from person to person; persistent phage can be linked to abundant host bacteria in the gut (Shkoporov et al., 2019). Phage may play key roles in regulating bacterial abundance in the microbiome. If bacteria with CRISPRs that render them resistant to these phage are consumed, their resistance could potentially spread to the commensal bacteria via horizontal gene transfer (Godde and Bickerton, 2006; Horvath et al., 2009), leading to dysbiosis. Another role for phage in the human microbiome is as an antibacterial layer that protects mucosal surfaces (Barr et al., 2013; Barr, 2017). If phage play this role in the gut of healthy individuals, consumption of bacteria resistant to these phage could also lead to dysbiosis and potential bacterial infiltration of epithelial cells (Ogilvie and Jones, 2015). Data gathered in this CURE could help illuminate the potential for these types of interactions by identifying the bacteriophages that commonly consumed lactic acid bacterial strains are immune to.

### Potential Student Outcomes

In this methods article we provide guidelines to instructors who would like to incorporate this CURE as a module in a microbiology lab course. A brief, 2–3 weeks module like this can be an accessible way to begin to bring open-ended research into an existing class, and the specific topic should be of broad interest to students. Students who can see connections between the science they are learning and the real-world perform better and are more likely to persist in science (Cromley et al., 2016), particularly if they can see how the science may be personally relevant to their lives, which may be especially important for underrepresented minorities (Hurtado et al., 2010). By connecting hands-on research with a question relevant to the everyday lives of students and their communities (what people eat and how it can affect them), this module aims to stoke students’ interest and engagement with science, and contribute to a reduction in achievement gaps.

How do we hope to achieve these goals? This module brings authentic research to students as part of their normal curriculum. Though there are various frameworks for evaluating the authenticity of research experiences (reviewed in Rowland et al., 2017), we have taken advantage of the simplicity of the CUREnet five dimensions framework (Auchincloss et al., 2014) and have mapped how this module embodies each dimension in Table 1. We have only begun to assess the impact of this module on students. However, in the future we hope to assess cognitive, affective, and psychosocial outcomes, and have suggested instruments to do so in the discussion.

## Methods for Implementing Module as Part of a Course

**FIGURE 1:**
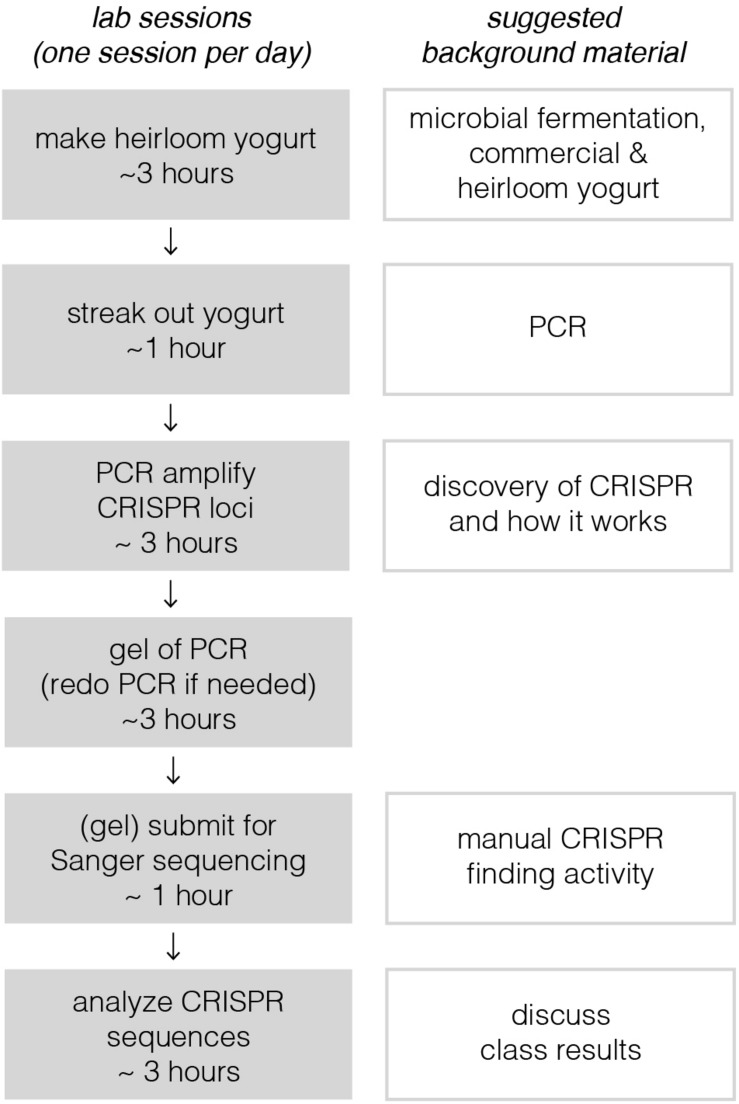
Breakdown of lab sessions and suggested lessons for downtime or lectures in between labs. On the left, each lab session, constrained by a bubble, is designed to be carried out on a single lab day. Sessions are broken up to allow for overnight incubation steps or when tasks would take too long for students to complete in a 3-h window. Depending on course frequency, these six sessions, occurring on six separate lab days, would likely take 2–3 weeks at most institutions. This could in principle be shortened to as little as four consecutive days. With a full-day lab session, the third, fourth, and possibly even the fifth sessions could be combined. Timing for the last session depends on how quickly sequencing results could be returned; there are many same-day and next-day services available.

Implementation of this module requires six active lab sessions, taking a maximum of 3 h each (see Figure 1). Some of the sessions do not require all 3 h, so they could be combined with lectures to provide key background information or discussions where students share results with one another. The following sections provide a general lab guide for instructors, covering key considerations and pitfalls to avoid for each step of the module. Detailed, step-by-step instructions for students are provided in the supplementary manual, which also includes background information and explanation of the protocols. To expedite time in the lab, we usually pre-aliquot the reagents required by each group of students; we usually provide a little extra volume than what is specified in the Supplementary Material.

### Producing Heirloom Yogurt

Heirloom yogurt contains an undefined mixture of microbes originally derived from the environment. If instructors have access to a heirloom yogurt culture at home, or from a friend or relative, it could certainly be used for this module and propagated indefinitely. However, not everyone has access to a home-grown heirloom culture. Fortunately, the popularity of home-fermentation has resulted in some commercially available heirloom cultures.

In our implementation of the class, we use two heirloom yogurt starters available from the company Cultures for Health (2020). It is important to select starters that are actually labeled (in the product name or in the description) as “heirloom;” the company also sells conventional, defined-species starters (which they somewhat confusingly sometimes refer to as “traditional” in that, for most of the past century, defined-strain yogurts were the only ones commercially available in the United States). Starters are supplied as a dried powder; we pre-aliquot 0.8 g of the powder (1/10th of the packet) into individual microfuge tubes so the students can then easily tap out the entire contents of the microfuge tube into 100 mL of scalded milk. (To scald the milk, it is poured into individual Pyrex bottles with loose lids, brought to 82–85°C in a water bath and held for 10 min, then stored at 4°C. Prior to class, it is pre-warmed to 43°C).

### Isolating Potential *S. thermophilus* Colonies

Both heirloom and commercial yogurts may be made with a wide variety of lactic acid bacteria, however there are two species that are most prevalent. These are *Streptococcus thermophilus* and *Lactobacillus bulgaricus*. They have a symbiotic relationship in yogurt cultures (reviewed in Aryana and Olson, 2017), and all commercial yogurt sold in the United States is required to be made with at least these two species. To facilitate comparisons between different yogurts, the module focuses exclusively on these two strains. While we have amplified CRISPR regions from *L. bulgaricus*, we have had more success with *S. thermophilus*, so the manual focuses only on it. Since the yogurts may contain other bacterial species, students start by producing t-streaks of either commercial or heirloom yogurts to isolate colonies for screening. In our implementation of the class, we have students work in groups of four, with one pair of students focusing on the heirloom yogurt and one pair working on the commercial. Commercial yogurts are obtained from the grocery store. Because yogurt is semi-solid and difficult to streak out, we recommend that students make at least three streak plates from each type of yogurt.

In the module presented here, we rely on brute-force PCR screening (next section) of many colonies as the way to simultaneous identify whether an isolate is *S. thermophilus* and also amplify its CRISPR loci. An optional way to extend this portion of the project would be to add a few lab sessions where students first screen candidate colonies using conventional approaches to differential identification. In previous implementations of the course, we have had students microscopically examine cell appearance, arrangement, and Gram characteristics, and conduct fermentation tests (in Durham tubes, with lactose, mannitol, or sucrose) and temperature sensitivity tests (at 45 and 15°C) to help discriminate *S. thermophilus* from other common lactic acid bacteria. (After the microscopic examination, students re-streaked their candidates so they wouldn’t run out of cells). While this introduces students to traditional methods of microbial identification with Bergey’s Manuals (Bergey and Holt, 2000; Vos et al., 2009), we have often found that the tests can be inconclusive and many students don’t identify *S. thermophilus* until they screen new colonies during PCR anyway. The physiological approach to identifying bacterial unknowns has been used extensively for many decades as an inquiry-driven approach to college microbiology lab courses, and has been thoroughly described elsewhere (Callery et al., 1980; Ziser, 1983; Deutch, 1994; Wagner and Stewart, 2000; Johnson and Case, 2019), so here we limit our description to the PCR screen.

### Amplification of CRISPR Loci

Students carry out colony PCR with primers that bind to conserved sequences just outside of the CRISPR repeat/spacer array. Colony PCR avoids time-consuming genomic DNA extraction, and the suggested schedule (Figure 1) affords time for a “back up day” if student PCRs fail in their first attempt. If a student’s positive control works, but they don’t get any bands from their colonies, these colonies may not be *S. thermophilus*, and they should try new colonies. If the positive or negative control fails, students should have a discussion with the instructor about what may have gone wrong, and they can either pick new colonies or retry the initial, if there is enough material left. (For the “back-up” day, since it is hard to predict how many students will need to set up a second PCR reaction, we recommend against pre-aliquoting reagents. Instead, we just keep a few stock tubes on hand for the groups that need them).

The forward primer, ST1_fwd, is 5′-TGCTGAGACAACCTAGTCTCTC-3′, and the reverse primer, ST1_rev, is 5′-TAAACAGAGCCTCCCTATCC-3′. These are the primers “yc70” and “CR1-rev” from Horvath et al. (2008), renamed here to avoid student confusion. Four different CRISPR loci have been detected in *S. thermophilus* genomes; these primers are specific for the CRISPR1 locus (Horvath et al., 2008; Barrangou et al., 2013). Unlike the other loci, which are completely absent in some *S. thermophilus* strains, the CRISPR1 locus appears to be ubiquitously present amongst *S. thermophilus* strains, while simultaneously harboring the most diversity in its individual spacer sequences from strain to strain (Horvath et al., 2008). A lab-grown culture of *S. thermophilus* (available to purchase from ACCT), serves as the positive control.

Students determine the size of their CRISPR amplicons through gel electrophoresis. Since instructional labs utilize varying manufacturers of electrophoresis chambers, power supplies, and gel imaging instruments, we have left the accompanying protocol for this section intentionally vague. As a safety consideration, we do suggest using SYBR safe and blue LED illumination instead of ethidium bromide and ultraviolet light; however, either option will work. Be sure to remind students to save their PCR products; this is what they will submit for sequencing in the next step.

### Sequencing of CRISPR Loci

Once each group of students has a successful commercial and heirloom amplicon, they can submit them as templates for conventional Sanger sequencing. The ST1_fwd primer is used as a sequencing primer, and it is sufficiently upstream of the spacer array so that the start of the array can be accurately sequenced. Different sequencing service providers will have different specifications for the volume and concentration of template and primer. We use a service provider that will carry out PCR clean-up (removal of excess primers, protein, and dNTPs) for a small additional fee, but this could also be carried out by students if time allows.

It is important to note that the typical length of a Sanger sequencing run may not be sufficient to cover the entire amplified CRISPR array. In our implementation of the class, we have students focus on the 5′ end of the array, since this region reflects the most recent history of virus exposure (new spacers are added to the 5′ end of the array (Barrangou et al., 2007). However, if time and coincidence with course learning outcomes allows it, students could use a primer walking strategy to sequence the entire array, using their first sequencing results to design a forward sequencing primer corresponding to a spacer (not a repeat) about 100 bp from the 3′ end of their sequence.

### Characterization of Phage-Derived Spacers

Once students have their sequences back, they will identify the individual spacer sequences. This can be done manually simply by looking for stretches of sequence that seem to be repeated, then flagging the variable regions in between. I have provided an in-class activity (Supplementary Material) where students are challenged to do that using two common lab strains of *S. thermophilus.* However, to ensure reproducibility and make things a little less tedious for the students, for the actual results from the commercial or heirloom yogurt we have students use a bioinformatics tool, CRISPRCasFinder, that will identify repeat regions and create a list of spacers automatically (Couvin et al., 2018, available as a web-based tool^1^).

The CRISPRCasFinder web tool requires sequences in the fasta format. Many Sanger sequencing services provide output in the ‘.seq’ format. It is easy to convert the files in a text-editor (by adding a “>sequencename” header line), and there are also web tools like EMBOSS seqret^2^ that can perform the conversion.

Once spacers are identified, students can use the NCBI’s web-based BLAST to search for matches in its database^3^. It is important for students to pay attention to the organism names and focus on viral matches, as many of the top hits may actually be coming from the CRISPR arrays of closely related *S. thermophilus* strains. Some spacers may not return any viral matches; these are derived from new viruses whose sequence is not in the database.

Once students have a list of their spacers and a list of the amplicon lengths for the commercial and heirloom isolates, the intellectual work of interpreting the results comes in. Students can perform *t*-tests on the amplicon sizes (derived from the gel) to see if there is a statistically significant difference between commercial and heirloom samples in terms of the number of spacers. (Since spacers and repeats are regular lengths, the amplicon length is an effective proxy for the number of spacers). Students can also research the viruses their bacteria have immunity against to learn more about them: Where are they commonly found? What is known about their normal function in microbial ecosystems? Do they have any connection to the fermented food industry? Is there anything known about their potential role in human health? Sometimes there is not much information about a particular virus, but even looking up information on who submitted a virus genome (for example, a yogurt manufacturer), can provide insight. These research questions will require students to perform literature searches, read scientific journal articles, generate hypotheses and construct arguments for them with evidence. There are many possible ways students could demonstrate how they have researched these questions and how they interpret their results (lab reports, short essay questions on a worksheet, posts in a discussion forum, group presentations). In our implementation of the module, we explicitly (via a rubric) directed students to explore specific research questions in the discussion section of lab reports (Table 2).

**TABLE 2:** Guidelines for student exploration of data.

	Guidance used for student exploration and interpretation of data
1	Interpret 16S data in terms of how heirloom different from commercial and any unexpected results
2	Interpret meaning of class-wide results for difference in the CRISPR array size in commercial vs heirloom
3	Speculate on whether results do or do not have implications for human health (discuss general implications from class data as a whole AND at least one specific implication of one of the particular viruses your strains are immune to)
4	Discuss limitations of this project and ways to improve the method
5	Suggest future experimental or literature or database research directions

*To earn full credit, students needed to include all items in the discussion section of their lab reports. (Full lab report rubric available in Supplementary Material).*

## Results

### Implementation of Module in Undergraduate Lab Course

This module was incorporated into a redesigned upper-level Microbiology Laboratory course taken primarily by juniors and seniors. The module has been taught by three different instructors (including the author) since it was first developed in 2019, with eight offering of the class reaching ∼450 students (in person). In Spring 2020, two sessions of the class were held remotely for an additional ∼100 students, using a modified version of the module with analysis portion only, relying on data generated by previous classes.

Incorporating new activities into large-enrollment lab courses can be difficult, since there are often many different people involved. In our implementation of the course, there are multiple instructors teaching separate sections of the class every quarter. Multiple graduate instructional assistants (1 per 24 students) help lead the labs, and instructional laboratory support staff help prepare and aliquot reagents. To ensure that everyone was on the same page, the lead instructor for the course (the author) held weekly planning meetings with the entire instructional team. Additionally, a scale-up approach was taken to deploying the course — it was first piloted with a single instructor (again, the author) and small group of 24 students before expanding it to every section.

The module has been used to successfully gather data in every offering of the course. Sometimes, individual students may not be able to get PCR amplicons from their yogurt despite sampling multiple colonies; one possible reason is that *S. thermophilus* may have been present at particularly low-abundance in their sample. One potential way to improve the yield of *S. thermophilus* colonies would be to switch from MRS media to a more selective media, like M17 (Shankar and Davies, 1977). In cases where individual students are unable to obtain an amplicon, we have been able to redistribute amplicons from groups that had multiple successful PCR bands. In CURE modules that incorporate open-ended research, it is important to allow for the possibility of failure, and have back up material for analysis. We make sure not to look for specific or expected results as part of our graded assessments — instead we focus on student’s understanding of how results were generated and their ability to interpret them and make scientific arguments. When we do want to assess technical skills, we do so with small practical exams not tied to the main research module.

### What Was Learned About the CRISPR Loci in Yogurt-Derived *S. thermophilus* So Far?

Data collection and analysis is ongoing, but the size of CRISPR1, as measured by the amplicon size, varies considerably, from as small as 0.8 up to 4.0 kb. The loci tend to be bigger in the commercially derived strains, however, students do not always find that this difference is statistically significant. This may be due in part to errors in estimating band size — students estimate it “by eye” using DNA ladders. In the future we will have students fit a trendline to the distance traveled by each band in that ladder, and use that and the distance of their amplicon to more precisely calculate the size. Alternatively, primer-walking or a long-reads approach like nanopore sequencing could be used to sequence the entire array and more precisely measure its length from first to last spacer. Another potential factor is that students only used the amplicon size data generated by the other groups in their section (∼48 students, or 12 groups); and it is possible this is too small a sample to see significance. A third possibility is that a key difference between commercial and heirloom yogurts may not be in the number of phage they are resistant to, but in the phylogenetic diversity of those phage, which is another item students could explore. Going forward, we would like to incorporate more systematic analysis of the accumulated data from successive offerings of the course; this will also allow students to get a better sense of how science is an iterative process.

### What Did Students Learn From Participating in This Module?

An exploratory assessment of student knowledge and attitudes before and after completing the CRISPR module was conducted during one quarter of the course. In a brief pre- and post-module survey, students were asked to rate their agreement with several statements about yogurt, probiotics, the human gut microbiome, and their attitudes toward commercially produced and genetically modified food (shown in Table 3). Some items were based on an instrument used to assess healthcare professionals’ conceptions of probiotics (Wilson and Whitehead, 2019).

**TABLE 3:** Student agreement with statements from exploratory survey.

**Statement *responses were strongly disagree (0), disagree (1), neutral (2), agree (3), and strongly agree (4)***	**Pre-score**		**Post-score**			
	***Mean***	***SD***	***Mean***	***SD***	***Wilcoxon W***	***P***
1. Eating foods or supplements with certain types of bacteria can have a positive impact on human health.	3.15	0.795	3.27	0.761	24.00	0.430
2. In order to have any impact on human health, microbes must be alive at the time they are consumed.	2.45	1.063	2.85	1.093	49.00	0.055
3. In order to have any impact on human health, microbes must stay alive for at least part of the time they spend in the digestive system.	2.70	0.847	2.73	1.039	88.50	0.909
4. Commercially produced yogurt is safer than homemade yogurt.	2.15	0.906	2.12	1.083	133.50	0.825
5. Bacteria are used to produce yogurt.	3.33	0.777	3.88	0.331	8.50	< 0.001*
6. All yogurt sold in stores contains bacteria.	2.73	1.098	3.48	0.906	8.00	< 0.001*
7. Some yogurt sold in stores contains living bacteria.	3.18	0.0.584	2.91	1.208	135.00	0.251
8. All yogurt sold in stores contains living bacteria.	1.94	1.029	2.82	1.211	60.00	0.002*
9. We use this statement to discard the surveys of people who are not reading the questions. Please select “Agree” for this question to preserve your answers.						
10. I am comfortable eating food developed in a (food-grade) laboratory.	3.06	0.747	3.30	0.728	26.50	0.084
11. I am comfortable eating genetically modified foods.	3.03	0.770	3.18	0.769	28.00	0.178
12. Natural foods are safer than commercially produced foods.	2.45	0.833	1.97	0.728	170.00	0.011
13. Scientists have a good understanding of how the human gut microbiome influences health.	2.30	0.984	2.06	1.144	107.50	0.334

Deidentified student response data was analyzed as follows. Only students who completed both the pre- and post-survey on time were included, and students who did not respond to all items were removed. Students who did not correctly respond to the control statement (item 9) were removed, and this item was not used in subsequent analysis. For each item, students selected “Strongly Disagree,” “Disagree,” “Neutral,” “Agree,” or “Strongly Agree;” these were converted to scores of 0, 1, 2, 3, or 4, respectively. Pre- and post- scores for each student were compared using a Wilcoxon signed-rank test (non-parametric, paired, *n* = 33). Statistics were computed in jamovi (The jamovi project, 2020). Mean scores and standard deviation are shown, and items with a significant difference in response distribution (Bonferroni corrected α of *p* < 0.004) are starred (*).

Quantitative analysis of the survey data showed that, for most items, there was no significant change in student response (Table 3). Among items which did show a significant shift in student response, items 5 and 6, “Bacteria are used to produce yogurt” and “All yogurt sold in stores contains bacteria” show an increase in students correct understanding of the role microbes play in the everyday production of food in the real world. There was also a significant increase in item 8, “All yogurt sold in stores contains living bacteria,” though this item perhaps reflects student perception, rather than underlying knowledge, since some yogurt is heat-treated to kill off the bacteria prior to sale.

Students were also asked to respond to the open-ended question “What was the most important thing you learned from the microbes and industry (yogurt) module?” Selected responses are shown in Table 4. Some students (student 1 and student 2 in the table) reported that it was easier to understand CRISPR when they see where it comes from, despite having learned about its use in genetic engineering in their other courses. This suggests that incorporating the basic microbiology of modern applied techniques may help students better understand them. Other students (student 3 and student 4 in the table) emphasized that this module gave them an increased awareness of microbiology’s role in their everyday lives. (All responses were collected with approval of UC San Diego Institutional Review Board).

**TABLE 4:** Selected student responses to open-ended question on what they learned from module.

	What was the most important thing you learned from the microbes and industry (yogurt) module?
Student 1	“*I had a much better understanding on how CRISPR arrays works and how CRISPR is prevalent in common things and not just in a lab. if that makes sense.*”
Student 2	*“The most important thing I learned was the mechanism of CRISPR, as I had learned about CRISPR in other science classes but never learned where it came from and exactly how it works, which could be crucial as it appears the age of genetic modification is inevitable.”*
Student 3	*“How we can analyze the CRISPR loci to determine viruses that have infected yogurt bacteria and how this might give insight to how it affects our health and which kind of yogurt we consume.”*
Student 4	*“That commercial yogurt is ‘vaccinated’ against a wide variety of phages in order to resist more attacks.”*

It is likely that there are other underlying gains in student understanding and attitudes toward science not addressed by this exploratory survey. Future assessments of the impact of this module on content understanding and attitudes will feature items more specifically connected to the module, and will use Likert scales with multiple items to assess each content area to improve accuracy.

Ideally, our assessment would measure broader impacts of the module on student understanding of the nature of science and the psychosocial attitudes associated with persistence in science. The extent to which students perceive dimensions of research could be measured using instruments such as the Laboratory Course Assessment Survey (Corwin et al., 2015). Student understanding of the nature of science could be measured with an instrument like the Student Understanding of Science and Scientific Inquiry (SUSSI) survey (Liang et al., 2008), and psychosocial outcomes could be measured with instruments like Persistence in the Sciences (PITS) Survey (Hanauer et al., 2016). This module was implemented as part of a full redevelopment of our microbiology lab curriculum, which included other substantial changes and a second CURE module in addition to this module. When comparing the revised version of the course to previous versions, it would be impossible to distinguish the effects of the CRISPR module from effects caused by the other changes. We hope that future assessments of this module can include these broader outcomes, with a particular focus on whether the module has a positive impact on students from underrepresented groups.

## Discussion

This article shares a module that could be deployed to bring more research into existing classes. The module asks a research question with potential bearing on human health, and the research question is ideally suited for a CURE, in that it benefits from collection and analyses of many data points that would be difficult to automate. Here, broad lessons learned from our initial offerings of the course, as well as potential future directions, will be discussed.

### Lessons Learned

We had students share data with instructors and each other through shared, editable, online spreadsheets (in google docs). They then drew on this data when writing up lab reports. A drawback of this was that students didn’t always supply all of the requested information, and the data sheets experienced a kind of “format creep” as edits were sometimes made to the underlying structure, making it difficult to swiftly extract specific fields for later analysis. Going forward, a more robust data collection method will be used, perhaps making use of online-forms with questions that must be answered in order to earn full credit on assessments. Not only will this help ensure data is reported correctly, it should make it easier to assemble and compare data from multiple classes.

Another lesson was that it was difficult, as a class, to synthesize everyone’s data for a holistic analysis. In lab reports, students focused mostly on their own results, especially for the viral matches to the CRISPR spacers in their strain. In the future, there will be more time built into the class itself for discussion of the overall results. Even then, it may be hard to systematically summarize all of the data generated. Since one of the goals of the CURE is to contribute to science, this summative analysis is an important component. In the future, graduate student researchers may be recruited to help with this analysis, or it could be used as the basis of a data-focused undergraduate seminar course.

Even if additional analysis by other students is required, we want to make sure that every student participating in the module has a sense of agency while analyzing results. So far, we tried to accomplish that mainly through student exploration of research questions specific to their CRISPR loci (Table 2), as large class sizes limit the number of alternative materials we could provide for students if we asked them to design their own experiments. There are however, opportunities to incorporate more student agency. Students could choose the species and/or particular CRISPR loci (if a species has more than one), and design their own primers to amplify that CRISPR region. Without changing the wet-lab protocol, it could also be possible to give students more agency with a less prescriptive final assessment — rather than strictly following a lab report rubric, students could propose and pursue a literature and/or bioinformatic research question of their own choosing, which could increase their sense of project ownership.

### Online Options

In Spring 2020, with the global pandemic forcing remote instruction, students carried out the analysis steps with data from a previous quarter. In retrospect, this may have been a missed opportunity, as NCBI’s microbial genomes^4^ currently has genomes for 70 different *S. thermophilus* strains. Students could copy the CRISPR1 locus (or make a virtual amplicon based on the primers) and then characterize the CRISPRs of these strains. Students could even characterize other CRISPR loci in *S. thermophilus* or the CRISPR loci of other lactic acid bacteria involved in fermentation. Carrying out the analysis on public data sets is a viable option for labs forced online, or for instructors wishing to incorporate this module into a lecture course or a dry-lab (bioinformatics) course.

### Future Directions

There is a certain tension inherent in sharing a specific CURE module with the educational community. The goal is to get more students involved in research and to recruit contributors of additional data, so we aim to show that has been successful. However, for the CURE to be a genuine research opportunity, the scientific outcome of the research question can’t already be solved, and indeed, to make the CURE sustainable over a period of more than a few years, should be something open to continuous addition and refinement. This CURE certainly has not yet fully resolved its central questions of what immunity is present in strains used for food production and what the potential consequences of that immunity are for human health. The first goal is to create a catalog of CRISPR spacers found in *S. thermophilus* used in yogurt production; the listings in this catalog could be expanded and validated by additional contributions, and could be broadened to include other species and other types of fermented food. Ultimately this catalog could be used a resource to better understand potential interactions of microbes we consume with the human gut microbiome. To that end, the plan is to create an online public repository for this data. Queries from instructors and researchers interested in participating are welcome.

## Data Availability Statement

The raw data supporting the conclusions of this article will be made available by the authors, without undue reservation.

## Ethics Statement

The studies involving human participants were reviewed and approved by UC San Diego Human Research Protections Program Institutional Review Board (opt-out consent forms were distributed in accordance with approved research plan; participants who opted out were removed and data was de-identified by the university before analysis began). Written informed consent for participation was not required for this study in accordance with the national legislation and the institutional requirements.

## Author Contributions

KP developed this course module, wrote instructional materials for it, collected and analyzed assessment data, and wrote the manuscript.

## Conflict of Interest

The author declares that the research was conducted in the absence of any commercial or financial relationships that could be construed as a potential conflict of interest.

## Footnotes

1 https://crisprcas.i2bc.paris-saclay.fr/CrisprCasFinder/Index

2 https://www.ebi.ac.uk/Tools/sfc/emboss_seqret/

3 https://blast.ncbi.nlm.nih.gov/Blast.cgi

4 https://www.ncbi.nlm.nih.gov/genome/microbes/

## Supplementary Material

Supplementary Data Sheet 1 CRISPR-finding by hand activity.

Supplementary Data Sheet 2 CRISPR-finding by hand activity answer key.

Supplementary Data Sheet 3 Lab Manual w. Background and Protocols.

Supplementary Data Sheet 4 Lab report rubric.
