# Supplementary material for: There’re CRISPRs in My Yogurt: A Discovery-Based CURE at the Intersection of Industrial Food Production and the Human Microbiome
Source: Front Microbiol. 2020 Oct 22;11:578737. doi: 10.3389/fmicb.2020.578737 (PMC7642981; doi:10.3389/fmicb.2020.578737)
Supplement: Supplementary Data Sheet 2 — CRISPR-finding by hand activity answer key. [file Data_Sheet_2.PDF]

LMD-9 (648,892-650,349)
primer binding sites highlighted, spacer: GTTTTTGTACTCTCAAGATTTAAGTAACTGTACAAC

5’ACTGTGACACCTTATTTATTGACAAAAATGCTGAGACAACCTAGTCTCTCACTTGTTGATTCAGTAATATTGAATAT
CCTAAATCAGTTGTTTCATTTTAGTTACCGTATAAGATATTTACAAAAATCTGATGAAAAACTTTTACAGAAATTTTTA
GAAAGTAAGGATTGACAAGAACAGTTATTGATTTTATAATCACTATGTGGGTATGAAAATCTCAAAAATCATTTGAGGT
TTTTGTACTCTCAAGATTTAAGTAACTGTACAACATGATGATGAAGTATCGTCATCTACTAACGTTTTTGTACTCTCAA
GATTTAAGTAACTGTACAACCTTCACCTCAAATCTTAGAGCTGGACTAAAGTTTTTGTACTCTCAAGATTTAAGTAACT
GTACAACATGTCTGAAAAATAACCGACCATCATTACTGTTTTTGTACTCTCAAGATTTAAGTAACTGTACAACGAAGCT
CATCATGTTAAGGCTAAAACCTATGTTTTTGTACTCTCAAGATTTAAGTAACTGTACAACTAGTCTAAATAGATTTCTT
GCACCATTGTAGTTTTTGTACTCTCAAGATTTAAGTAACTGTACAACATTCGTGAAAAAATATCGTGAAATAGGCAAGT
TTTTGTACTCTCAAGATTTAAGTAACTGTACAACTCTAGGCTCATCTAAAGATAAATCAGTAGCGTTTTTGTACTCTCA
AGATTTAAGTAACTGTACAACTAAAAACATGGGGCGGCGGTAATAGTGTAAGGTTTTTGTACTCTCAAGATTTAAGTAA
CTGTACAACACAACCAGCAAAGAGAGCGCCGACAACATTGTTTTTGTACTCTCAAGATTTAAGTAACTGTACAACTATA
ACACAGGTTTAGAGGATGTTATACTTGTTTTTGTACTCTCAAGATTTAAGTAACTGTACAACCTAGAAGCTCAAGCGGT
AAAAGTTGATGGCGGTTTTTGTACTCTCAAGATTTAAGTAACTGTACAACCTTTGAGGGCAAGCCCTCGCCGTTCCATT
TGTTTTTGTACTCTCAAGATTTAAGTAACTGTACAACAACTACCAAGCAAATCAGCAATCAATAAGTGTTTTTGTACTC
TCAAGATTTAAGTAACTGTACAACCTATAAGTGACAATCAGCGTAGGGAATACGGTTTTTGTACTCTCAAGATTTAAGT
AACTGTACAACATCAGTGCGGTATATTTACCCTAGACGCTAGTTTTTGTACTCTCAAGATTTAAGTAACTGTACAACAA
CAGTTACTATTAATCACGATTCCAACGGGTTTTTGTACTCTCAAGATTTAAGTAACTGTACAGTTTGATTCAACATAAA
AAGCCGGTTCAATTGAACTTGGCTTTTTAAAATACACGATAAACATAAGGATTGTCAGGCTGACTAACCTCTTTAACCT
CAGTCAAATTAAGGATAGGGAGGCTCTGTTTAAGGTTT3’

LMG-18311 (629,691-632,244)
primer binding sites highlighted, spacer: GTTTTTGTACTCTCAAGATTTAAGTAACTGTACAAC

5’AAAAATGCTGAGACAACCTAGTCTCTCACTTGTTGATTCAGTAATATTGAATATCCTAAATCAGCTGTTTCATTTTA
GTTACCGTATAAGATGTTCTCAGACACCTGATAAGGAACTATTACAGAAATTTTTAGAAAGTAAGGATTGACAAGGACA
GTTATTGATTTTATAATCACTATGTGGGTATGAAAATCTCAAAAATCATTTGAGGTTTTTGTACTCTCAAGATTTAAGT
AACTGTACAACGAGCTACCAGCTACCCCGTATGTCAGAGAGGTTTTTGTACTCTCAAGATTTAAGTAACTGTACAACCG
TTCCTTTTTTCAAGGTAATCTTTGAAAGGTTTTTGTACTCTCAAGATTTAAGTAACTGTACAACAAGTCCGTAAGCACC
AGTTCCAATCGTCATGTTTTTGTACTCTCAAGATTTAAGTAACTGTACAACTTGAATACCAATGCCAGCTTCTTTTAAG
GCGTTTTTGTACTCTCAAGATTTAAGTAACTGTACAACAACCTCATACATGGGGAAAATTGGTAAGTAGTTTTTGTACT
CTCAAGATTTAAGTAACTGTACAACTAACTTCATTAGTGTAGTTGTAATTAGCATGTTTTTGTACTCTCAAGATTTAAG
TAACTGTACAACTTAGCTACCCAAATATCTTCTGTTTTCCAAGTTTTTGTACTCTCAAGATTTAAGTAACTGTACAACG
AGTTTTCAATATTGGCACAGGAGACAATTGTTTTTGTACTCTCAAGATTTAAGTAACTGTACAACTGATACTATTTTAG
TCAGATATGAAATATCGTTTTTGTACTCTCAAGATTTAAGTAACTGTACAACTCATCAATGTTTAAAGCCCAACAATAC
ATGAGTTTTTGTACTCTCAAGATTTAAGTAACTGTACAACTAGATTTAATCAGTAATGAGTTAGGCATAAGTTTTTGTA
CTCTCAAGATTTAAGTAACTGTACAACAGGAAAATAGCATGAGCGTACAACAATCTAGTTTTTGTACTCTCAAGATTTA
AGTAACTGTACAACTGTCTATCACGCTTCCTAAGTGCATGAAAAGTTTTTGTACTCTCAAGATTTAAGTAACTGTACAA
CATGTCACCAATCACTAAAGAACCTACGCTGGTTTTTGTACTCTCAAGATTTAAGTAACTGTACAACAACATCTTCCTC
TCCGATTGCAAATAGTGCGTTTTTGTACTCTCAAGATTTAAGTAACTGTACAACCATATTTGGTGCCCGTTCGATAAAG
AGTAGTTTTTGTACTCTCAAGATTTAAGTAACTGTACAACCATTAAATCGCTTGAAGCAGACATTGAAGCGTTTTTGTA
CTCTCAAGATTTAAGTAACTGTACAACGACTTATCTTGGAAGGTAGTGAAGGCACTTGTTTTTGTACTCTCAAGATTTA
AGTAACTGTACAACTCCTTGCCATCTGCACTGTAAGCCCAAGCAGTTTTTGTACTCTCAAGATTTAAGTAACTGTACAA
CTAGTACGCATAATCAATTCATCAAGCTTGAGTTTTTGTACTCTCAAGATTTAAGTAACTGTACAACGTAGTGACCCAA
AATTCTATGACCTTGAAAGTTTTTGTACTCTCAAGATTTAAGTAACTGTACAACAGATTGTGGTGCTTACGGAAAATTC
CTTGTGTTTTTGTACTCTCAAGATTTAAGTAACTGTACAACTGGCAAGAAGTGTAAGAGATGCAATGGATAGTTTTTGT
ACTCTCAAGATTTAAGTAACTGTACAACTTTATTATCATTATTCTTCTTCCCAAGCGTGTTTTTGTACTCTCAAGATTT
AAGTAACTGTACAACTTTTATAGAATTTGGTGGTGAACTTTTTCAGTTTTTGTACTCTCAAGATTTAAGTAACTGTACA
ACAATGGGTCACAGATTGCCATAATAAGGAGGTTTTTGTACTCTCAAGATTTAAGTAACTGTACAACCCGAGGTCACTT
TAGAACCCACAAAATAAGGTTTTTGTACTCTCAAGATTTAAGTAACTGTACAACATGAGAGAACACAGTATAGACCCTG
ATACAGTTTTTGTACTCTCAAGATTTAAGTAACTGTACAACCAGTATTAATGAGGTTTGGGTGGTCATTCCGTTTTTGT
ACTCTCAAGATTTAAGTAACTGTACAACCCATACTCTCTATCAGTTCATTTAATTCTTCGTTTTTGTACTCTCAAGATT
TAAGTAACTGTACAACTAATATGTCGCTCTACTGATTCCAAAACGGGTTTTTGTACTCTCAAGATTTAAGTAACTGTAC
AACATGAATTACATTCATGATTTTATCGAGTTTGTTTTTGTACTCTCAAGATTTAAGTAACTGTACAACCGTGCCATTG
TTTCGGTCGGACGTGGGCAGTTTTTGTACTCTCAAGATTTAAGTAACTGTACAGTTTGATTCAACATAAAAAGCCAGTT
CAATTGAACTTGGCTTTTTAAAATACACGATAAACATAAGGATTGTCAGGCTGACTAACCTCTTTAATCTCAGTCAAAT
TAAGGATAGGGAGGCTCTGTTTAAGGTT3’
