# Supplementary material for: There’re CRISPRs in My Yogurt: A Discovery-Based CURE at the Intersection of Industrial Food Production and the Human Microbiome
Source: Front Microbiol. 2020 Oct 22;11:578737. doi: 10.3389/fmicb.2020.578737 (PMC7642981; doi:10.3389/fmicb.2020.578737)
Supplement: Supplementary Data Sheet 3 — Lab Manual w. Background and Protocols. [file Data_Sheet_3.PDF]

“There’re CRISPRs in my Yogurt” - Background & Protocols

Module overview

Microbes have played a role in the preservation of foods for thousands of years. When certain types of microbes grow in a food or beverage (milk, grape juice, certain preserved meats, etc.), they change the properties of their surrounding environment to make it inhospitable to other microorganisms that might otherwise spoil the food or make it inedible. Lactic acid bacteria are a particular type of microorganism especially important for the dairy industry. These microbes break down the lactose carbohydrates in milk via a metabolic process called fermentation, producing lactic acid as a byproduct.

Traditionally, fermentation was carried out with ‘wild’ microorganisms that somehow made their way into dairy products and resulted in a pleasant tasting preserved product like yogurt. These cultures would be propagated by adding a small amount of the yogurt to fresh milk, and, if the composition of microbes was right, this process could be repeated to continually culture the same collection of microbes (or “community” of microbes) for years or even decades. Today, some yogurt is still made this traditional way, using cultures of mixed microorganisms that are called “heirloom” cultures.

However, the modern yogurt industry, which is responsible for most of the yogurts available in stores, relies instead on using defined mixtures, and only a few microbes, whose genus, species, and strain is known, are used in the fermentation. Because commercial yogurts only contain a few types of microbes, they usually can’t form the complex, self-perpetuating communities that heirloom cultures can. So commercial yogurt usually can’t be used to make more than a few generations of new yogurt, and yogurt companies inoculate their milk with fresh microorganisms every time they make it.

In this project, you’ll be comparing the microbial diversity of commercial and heirloom yogurts. Specifically, you’ll be taking a look at the immune system of one of the microbes you find (CRISPR sequences). Much like humans and our antibodies, microbes with a different history of virus exposure will have immune systems that carry a different record of the viruses they’ve met. You will compare these immune systems in microbe strains isolated from commercial and heirloom yogurts, to see if they are different, and to potentially reveal whether the commercial yogurt companies are ‘vaccinating’ their yogurt strains by pre-exposing them to different viruses.

Learning outcomes for module. *By the end of this module you will be able to...*

1. Make yogurt.

2. Explain how microbes that carry out fermentation can help preserve food.

3. Analyze CRISPR loci in yogurt microbes to better understand their history of virus exposure.

4 Appreciate the research and development behind the commercial dairy industry.

5. Think critically about the food supply and its possible influences on human health.

Making yogurt

Objective

The goal here is to make yogurt from an heirloom starter, which is an undefined mix of 'wild' microbes. Once you've made the heirloom yogurt, you will compare them to commercial yogurts purchased from the grocery store, based on simple physical characteristics. We are making our heirloom yogurt from scratch because you can't buy heirloom yogurts in the grocery store. Once you've compared the physical features of the yogurts, we will take a deeper dive into one way the strains in the heirloom and commercial yogurts might be different.

Lab session 1. Make yogurt from heirloom starters (~3 hours)

As a group, you will make two 'heirloom' yogurts. You'll start by adding the microbes to milk which has been scalded (previously heated to kill unwanted microbes) and pre-warmed. After you add the bacteria, you will initially incubate the milk at 43°C. All commercial yogurt sold in the United States is required to have two bacterial species: *Streptococcus thermophilus* and *Lactobacillus bulgaricus*. These bacteria are likely playing a role in the heirloom yogurts as well, but so are a wide variety of other bacterial species. In commercially produced yogurt, the initial high temperature would allow the thermophilic *S. thermophilus* (get it, it's in the name) to grow. After that, the yogurt is cooled to a lower temperature, which, allows other bacteria, like *L. bulgaricus*, take advantage of how *S. thermophilus* changed the environment, and start growing. The final frequencies of each type of bacteria depends on the starting amounts and the growth conditions, but there will be living bacteria in the final yogurt products.

Important notes

> ***safety point: DO NOT EAT THE YOGURT!*** *Yogurt is perfectly safe to eat when made at home or in a food preparation facility. However,* ***yogurt made in the lab is NOT safe to eat.*** *Although the bacteria themselves are generally considered safe, the containers, instruments, and surfaces in the lab may have previously come into contact with* ***hazardous microbes, hazardous chemicals****, or even* ***broken glass*** *fragments, and we don't want you to ingest those. Additionally, it is against regulations and could get us in trouble if* ***anyone*** *is caught eating* ***anything*** *in the lab.*

Materials

- 100 mLs of scalded whole milk (not ultra-pasteurized or UHT, scalded by holding at 82-85 °C for 10 minutes), pre-warmed to 43°C (1 bottles per group of 4)
- "Cultures for Health" heirloom yogurt starter, 0.8 g (1 per group of 4)
- pH strips
- plastic spoon

Protocol

1. Use sterile technique throughout this activity.

© Katherine Petrie, 2020 | Do not copy or distribute without permission

2. **Label** your bottle of milk. This milk has been scalded (heated at 85°C for 30 minutes) to kill any microorganisms that may have been present in the milk. Be sure to include your initials, and which starter culture you are using.

3. Lay out a pH strip on a kip wipe, and use a micropipettor to place a drop of the milk from your bottle onto the pH strip, making sure the square is covered. **Record the pH** of the milk.

4. **In each group, each pair of students** will prepare a different heirloom yogurt following the protocol below.

> - add all of the freeze-dried bacteria in the microfuge tube to your milk (just open the tube, tip it over your milk and tap). If you have trouble getting it all, you can use a micropipettor to take 1 mL of the milk and pipette it up and down in the tube, then transfer it back into the bottle.
>
> - use a plastic spoon to thoroughly mix the cultures in

5. Measure and record the **pH of the milk + starter cultures** using the same technique as in step 3.

6. Check to see if any curdling (thickening or coming together in clumps) occurred in the yogurt when you added the starter cultures by examining and recording the consistency.

7. Place your bottle at 43°C and note the time.

8. After the yogurt has been incubating for **two** hours, carefully take it out, and **measure the pH** again. At this point, **do not stir** your yogurt while you are examining it - just take a small sample from the top for the pH check. Has the yogurt started to set? If you tilt the bottle gently, does the yogurt come away from the side of the bottle as one mass or run like liquid? **Return the bottle to 43°C** when you are done making your observations.

9. After you have completed step 8, you are done with the yogurt for today. The yogurt will remain in the incubator, held at 43°C for another 8 hours (10 hours total). The yogurt will then be held at 25°C for 2 hours, then it will be stored at a refrigerated temperature (5°C) until the next lab session.

© Katherine Petrie, 2020 | Do not copy or distribute without permission

Lab Session 2. Compare commercial and heirloom yogurt. (~ 30 minutes)

Here you will compare the physical features of the different yogurts.

Important notes

safety point: (still) ***DON'T EAT THE YOGURT!***

Materials

heirloom yogurt from last time (1 per group)
commercial yogurt (1 per group)
pH strips
plastic spoon

Protocol

1. Use sterile technique throughout this activity.

2. Working with your group, collect observations about your heirloom yogurt and commercial yogurt. Note the **appearance** and **smell**.

3. If it looks like the whey has separated from any of your yogurts, use a plastic spoon to **stir** it back in until the yogurt returns to an even consistency (homogenized).

4. Determine the **consistency** of your yogurt by dipping a spoon in it and assigning one of the metrics below.

    - completely solid (protein fully coagulated, curds separated from clear liquid whey and can't be stirred back in)

    - thick (spoonful of homogenized yogurt flows slowly and drips off spoon in large globs)

    - thin (spoonful of homogenized yogurt flows more quickly and drips off the spoon in small globs)

    - liquid (spoonful of homogenized yogurt flows very rapidly, just like milk, with no evidence of thickening)

5. Measure and record the **pH** of each of the three yogurts. (Use a pipette tip or spoon to transfer a small amount to a strip set out on a kimwipe or paper towel). You may have to wipe off the yogurt after it sits on the pH strip for a few seconds to be able to read the strip.

© Katherine Petrie, 2020 | Do not copy or distribute without permission

Isolation of *S. thermophilus* strains & characterization of their CRISPR arrays

Objective

Here, you will do a focused examination of one of the signature yogurt species, *Streptococcus thermophilus,* to see how the species differs in commercial and heirloom yogurt. All yogurts contain *S. thermophilus,* but they may have different strains, or sub-types of the species, that vary somewhat in their DNA, particularly in the part of the genome that constitutes the bacterial immune system.

How will you know if your colonies are *S. thermophilus?* You will rely on a PCR screen using primers that are specific to unique sequences only found in *S. thermophilus.* These sequences are located in a region that is one of the most interesting places strains can differ, called the CRISPR loci. CRISPRs, or clustered, regularly interspaced, short, palindromic repeats, are found in the genome of many bacteria. They function as the bacteria's immune system, and work much like antibodies do in humans and other animals.

Antibodies are circulating proteins that 'remember' the shape of foreign bits of pathogenic microbes and viruses. When an infection occurs, they can recognize the invading pathogen and direct other components of the immune system to attack. CRISPRs work the same way, except instead of antibodies, the part that remembers invaders uses DNA and RNA. What infects bacteria? Viruses!

When viruses infect bacteria, they inject their DNA into the cell and, usually, hijack the cellular machinery to produce progeny viruses which burst out of the bacteria, killing it. However, in, some lucky bacterial cells the infection fails, and the bacteria survives. In these cases, if the bacteria has a CRISPR system, it will cut out a small piece of the virus's genome, and insert it into its own genomic DNA. These virus-derived sequences are what make up the CRISPR locus.

The sequences in the CRISPR locus are expressed as short molecules called guide RNA, and they partner up with an enzyme called Cas, and circulate around the cell. The next time a virus tries to infect the cell, if its genome has a sequence that matches the guide RNA, the guide RNA will bind to the viral genome, and the Cas enzyme will chop the viral genome into pieces, protecting the bacteria.

Over time, CRISPR loci can accumulate a record of the viruses that the bacteria has been exposed to, and is immune to. Because commercial and heirloom yogurts are produced in such different ways, we think the *S. thermophilus* strains in them will have very different CRISPR loci, corresponding to both different types and numbers of viruses. Our goal here is to characterize and compare CRISPR loci from both commercial and heirloom strains, and then to think about any potential implications for human health.

© Katherine Petrie, 2020 | Do not copy or distribute without permission

Lab Session 2. Isolate microbes from heirloom and commercial yogurts (~ 30 minutes)

The first step is to isolate individual microbes by diluting them on agar plates. After incubation, isolated colonies represent the bacterial progeny of a single cell, so you know that all the bacteria in that colony are members of the same strain. You will also take small aliquots of your heirloom and commercial yogurt to store as backup.

Materials

MRS agar plates (6 per group)
heirloom yogurt from last time (1 per group)
commercial yogurt (1 per group)
1.5 mL microfuge tube (2 per group)

Protocol

1. Use sterile technique throughout this activity.

2. From **each type** of yogurt (heirloom or commercial, NOT the lab strains), make **3 t-streak plates** on MRS agar. (We want to maximize the number of isolated colonies we get, so we're doing several t-streaks for each one)

3. Grow at 37°C, 24-48 hours.

4. As backup, transport a small amount (~1 mL) of heirloom yogurt and (~1 mL) of commercial yogurt to a 1.5 mL microfuge tube, label, and store at 4°C. Dispose of the remaining yogurt according to IA instructions.

© Katherine Petrie, 2020 | Do not copy or distribute without permission

Lab Session 3. Describe colony morphology, PCR amplify CRISPR loci (~ 3 hours)

Here you will examine several colonies from your commercial and heirloom yogurt streak plates. Your goal is to find three potential *S. thermophilus* each from the commercial and heirloom yogurt.

To screen the colonies, you will use PCR - polymerase chain reaction - to amplify the CRISPR region of your potential *S. thermophilus* candidates. The forward and reverse primers are designed to bind a little bit outside of the CRISPR locus, in a part of the *S. thermophilus* genome that is conserved, so that even if the different strains have different CRISPRs corresponding to different viruses, the entire region can be amplified.

To set up PCR you will combine your bacterial samples and primers with a mixture that contains a thermostable polymerase enzyme, dNTPs, and buffer.

Important Notes

science point: small samples of colonies! *Be sure that you do not sample the entire you want to make sure you have enough left over to go back to incase something goes wrong.*

science point: PCR! *PCR is very good at amplifying very small amounts of DNA. Use extra care when setting up your reactions to avoid unintentionally getting anything with DNA (bacterial cells, skin cells, etc.) in your reaction tubes.*

Materials

heirloom and commercial T-streaks from last time
Streptococcus thermophilus, strain LMG18311 (ATCC BAA-250) or strain LMD-9 (ATCC BAA-491)(1 per group of 4)
Q5 High-Fidelity 2X master mix (80 µL per group of 4) (NEB M0492)
5 µM ST1_fwd primer (20 µL per group of 4)
5 µM ST1_rev primer (20 µL per group of 4)
0.2 mL PCR tubes (12 per group of 4)
1.5 mL microcentrifuge tubes (10 per group of 4)
milliQ or molecular grade H_2O

Protocol

1. On the bottom of your T-streak plates from last time, circle 4 well-isolated colonies from each yogurt (heirloom or commercial) as the first candidates in your screen. Label each colony with a simple naming scheme (i.e. C1-C4 for commercial, H1-H4 for heirloom).

2. Describe the colony morphology of each of the colonies you circled.

© Katherine Petrie, 2020 | Do not copy or distribute without permission

3. With your partner, plan out your PCR. One pair of partners will set up the PCR reactions for the commercial candidates, and the other pair will set up the PCR reactions for the heirloom candidates. Each pair will have enough reagents for up to 4 candidates and 2 controls (6 total).

The following recipe lists the volumes required for a **single PCR reaction**, but you will not set up reactions individually - instead you will make a **master mix** that has everything in it except the individual bacterial samples. You should make extra master mix (the number of reactions you have + 1) to **allow for loss to pipette tips**. In addition to your candidates, you should also have a **negative control** reaction, and a **positive control** reaction. **Write out your recipe for your master mix**, and **have it checked by your instructor** before proceeding, but do not start making your master mix until everything else is ready.

recipe for single, 10 µL PCR reaction

component	final concentration	volume per 10 µL	volume in master mix (for 7, 10 µL reactions)
2X Q5	1X		
5 µM fwd primer	0.5 µM		
5 µM rev primer	0.5 µM		
diluted bacteria or H20	-	1 µL	add to each
MilliQ H20	-		

4. Prepare your test bacterial samples. Use sterile technique when you sample your plates. For the colonies, aliquot 20 µL milliQ each to 4 1.5 mL microfuge tubes. Then use a pipette tip to pick and swirl in a colony. Pipette up and down with an appropriately set micropipettor (at least ½ total volume) to mix well.

5. Prepare your control bacterial samples. Use sterile technique when you sample the positive control culture. Aliquot 18 µL milliQ each to a 1.5 mL microfuge tube. Add 2 µL of the *S. thermophilus* control strain. Pipette up and down with an appropriately set micropipettor (at least ½ total volume) to mix well.

6. Label your 0.2 mL PCR tubes. You should have one tube for each of your bacterial isolates, plus one tube for your positive control and one tube for your negative control (6 total). Be sure to label the tubes both on the top and on the side. Make sure you can tell the samples apart.

7. Assemble your master mix according to your **approved** recipe. Combine all components in a 1.5 mL microfuge tube **on ice** and pipette up and down with an appropriately set micropipettor (at least ½ total volume) to mix.

8. Aliquot 9 µL each of your master mix to your labeled PCR tubes, on ice. You should use a rack or keep tubes carefully closed to prevent ice-water from getting in the tubes.

9. Add 1 µL of your diluted bacterial sample to each of the appropriate tubes. For the negative control, add 1 µL H20.

© Katherine Petrie, 2020 | Do not copy or distribute without permission

10. For each reaction, pipette up and down with an appropriately set micropipettor (at least ½ total volume) to mix. Do not vortex anything containing the Q5, as it could damage the polymerase enzyme.

11. Set up the following protocol on a thermocycler

- heated lid on
- 95°C 5 min (this is required to break open cells)
- Cycle (98°C 10 sec, 64°C 20 sec, 72°C 90 sec) x 30 cycles
- 72°C 2 min

12. Right before you start the PCR reaction, transfer your tubes from the ice to the mini PCR thermocycler, and start the program.

13. When the PCR is complete, transfer the samples to the freezer.

© Katherine Petrie, 2020 | Do not copy or distribute without permission

Lab Session 4. Analyze PCR on agarose gel, try more PCR if needed (~ 3 hours).

The goal here is to check the PCRs from last time to see if you successfully amplified your positive controls and unknown stains of *S. thermophilus* from commercial and heirloom yogurt samples, and estimate how long your PCR amplicon is. You will also be able to set up another PCR if you need to screen more strains.

Important Notes

safety point: molten agar! *This is hot! Be careful not to spill it on yourself, and if you do, quickly remove your glove or stick it under water to avoid a burn.*

science point: small volumes! *After you load your gel, there will only be a small amount of liquid left in your PCR tube. Be careful not to lose this, as you will need at least 5 µL of it to sequence any successful PCRs.*

safety point: gel electrophoresis! *Most gel chambers are designed to only run a current when the lid is securely on. Nonetheless, use caution around the gel rig to avoid a shock.*

safety point: DNA dye! *The dye we use to illuminate the DNA in the gel is called SYBR safe, and, as they name suggests, is generally considered safe. However, caution should be used with any substance that sticks to DNA, so take care to not get the gel or gel running buffer on your bare skin.*

safety point: gel photography! *Follow your instructor's directions safely to take a photo of your gel.*

Materials

50 mL molten 1% agarose, held at 65 °C (2 per group of 4 students)
SYBR safe (5 µL per gel, see IA to have it added)
100 bp ladder (6 µL per group) (NEB, N3231)
1KB bp ladder (6 µL per group) (NEB, N3232)
6X purple loading dye (25 µL per group) (NEB, B7025)
1X TAE (enough to fill 2 gel rigs per group)
your PCR samples from last time
small tube rack
parafilm
gel electrophoresis rig, casting tray & comb, and power supply
blue transilluminator & orange filter
darken-able room or imaging box for photos
ImageJ (free software available at https://imagej.nih.gov/ij/, or other gel imaging software)

Protocol

1. Prepare your gel electrophoresis casting tray according to your instructors directions with combs that contain at least 8 wells.

© Katherine Petrie, 2020 | Do not copy or distribute without permission

2. Carefully get your molten agar and have your instructor **add 5 µL of SYBR safe**. Gently swirl to mix, then pour it into your prepared gel rid. If you have any bubbles, use a pipette tip to nudge them to the edges of your gel.

3. Wait until the gel is solidified. In the meantime, you can prepare your samples to load on the gel as follows:

- cut a piece of parafilm to cover 10 wells in a small tube rack, and gently press your figure over each well to make a small depression.

- Write down the order in which you will load you 6 samples (4 colonies, positive control, negative control) and two ladders (1 KB & 100 bp)

- aliquot 1 µL of 6X purple loading dye to each depression.

- add 3 µL of 1X TAE or milliQ H_2O to each depression

- add 2 µL of your PCR samples or ladder to each depression. **Save the rest of your sample!!!**

4. When the gel is solidified, carefully remove the comb and transfer it to the running chamber, then fill the gel running chamber with 1X TAE. Check that the TAE cover the gel by at least 0.5 cm. Make sure that the wells are on the side of the gel with the black (negative) electrode.

5. Clear out any air from the wells, using a small pipette tip.

6. Carefully load the gel. Put 6 µL in each well. Your samples on the parafilm may have evaporated a bit - this is fine, but you may want to gently depress the plunger so that there is no ‘air bubble’ at the end of your tip. Place the tip in the buffer just above the well opening - you can gently press against the opening of the well, but don’t stick the tip in very far, or you could tear the gel. Dispense your sample, and slowly lift the tip out before you release the plunger (otherwise you might suck your purple sample right back up). Repeat for the other samples, being careful not to bump the gel rig, as it could flush out the wells.

7. Once all wells are loaded, place the lid on and connect the electrodes to the power supply, again making sure that the DNA will run away from the negative electrode and towards the positive electrode (ask the instructor to check if you aren’t sure). Turn on the power supply, and set the voltage to 100 V, then push the running man to start the gel.

8. Run until the pink band is ½-¾ of the way down the gel (will take 20-40 minutes), then stop the gel, turn off the power supply, and remove the lid.

© Katherine Petrie, 2020 | Do not copy or distribute without permission

9. Carefully transfer your gel to a blue light box. You should put the gel directly on the lightbox (take it out of its tray). Watch the instructor for a demo on the best way to do this without breaking the gel.

10. Turn on the light box. In order to see the DNA bands, you will need an orange filter to block out the blue light, and see the fluorescent glow of the DNA dye (it absorbs blue light and fluoresces green) Hold the orange filter over the gel to see your results and discuss them with your partner.

11. Take a picture of your gel. To get the best visualization of the bands, you need to block out ambient light from the room. Follow your instructor's directions to get a good photograph of your gel.

12. Once you have a good image of the gel, discard the gel and TAE running buffer as directed by your instructor. Rinse the gel chamber, tray, comb, and dams, with DI water. Set them up on a paper towel to dry, and put them away before then end of class.

13. Open the photo of your gel in ImageJ, and label each of the lanes and every band in the ladder (indicate the size, in bp, of each band).

14. Answer the following questions.

What is the size, in basepairs, of each of your successful PCR reactions? (Use the ladders to help you estimate the size). Be sure to upload this information into the class datasheet as directed by your instructor.

Did any of your PCR reactions have problems? (i.e. missing band, faint band, multiple bands, too short band). If so, what do you think might have gone wrong?

15. Discuss your gel results with your group (and the instructor if you want) and decide whether you will run additional samples. You should have PCR bands from the positive control and **at least one successful PCR each from the commercial AND heirloom samples**. If you need to keep screening colonies, follow the directions below.

Materials

heirloom and commercial T-streaks
Streptococcus thermophilus, strain LMG18311 (ATCC BAA-250) or strain LMD-9 (ATCC BAA-491)
Q5 High-Fidelity 2X master mix (NEB M0492)
5 µM ST1_fwd primer
5 µM ST1_rev primer
0.2 mL PCR tubes
1.5 mL microcentrifuge tubes
milliQ or molecular grade H_2O

© Katherine Petrie, 2020 | Do not copy or distribute without permission

16. As a group decide how many colonies to screen and which plates your will use. Think about how many spots are available in the thermocycler and how many samples you can fit on a gel. Each master mix will need a positive and negative control to make sure your PCR worked.

17. Plan out, set up, and run your PCRs using the same general guidelines as last time. If your positive controls failed or if all of your samples failed the first time, talk the instructor about what might have gone wrong and strategize how to fix it.

© Katherine Petrie, 2020 | Do not copy or distribute without permission

Lab Session 5. Gel if needed, submit PCR for sequencing (~ 1 hour)

Here, if you ran a second PCR, you'll have time to run the PCR, and you will also select which amplicons you want to submit for sequencing. From the PCR, we have an idea of how long the CRISPR region is, but sequencing will allow us to see what viral sequences the *S. thermophilus* strains have in their immune system.

Materials - 2nd gel

50 mL molten 1% agarose, held at 65 °C (2 per group of 4 students)
SYBR safe (5 µL per gel, see IA to have it added)
100 bp ladder (6 µL per group) (NEB, N3231)
1KB bp ladder (6 µL per group) (NEB, N3232)
6X purple loading dye (25 µL per group) (NEB, B7025)
1X TAE (enough to fill 2 gel rigs per group)
your PCR samples from last time
small tube rack
parafilm
gel electrophoresis rig, casting tray & comb, and power supply
blue transilluminator & orange filter
darken-able room or imaging box for photos
ImageJ (free software available at https://imagej.nih.gov/ij/, or other gel imaging software)

Materials - submit for sequencing

your PCR reactions
8-tube PCR strip (1 per group)
for instructor: 5 µM ST1_fwd primer per sample (include in sequencing submission)

Protocol

1. If needed, follow the directions from last time to prepare and run a gel of your PCRs. Be sure to upload the length of any successful amplicons as directed by your instructor.

2. Decide which samples you will sequence. Remember, the goal is one commercial and one heirloom. If you have trouble deciding, ask your instructor for advice. If you don't have one of each, let the instructor know, and you may be able to sequence any extra's from other groups.

3. Sign up for a sequencing identifier, and label your tubes as directed.

4. Follow your instructor's directions to aliquot the samples you want to sequence to individual tubes. You may have to dilute your sample with milliQ H_2O to a particular volume. Double check that your tubes are fully closed.

6. Turn your samples directed by your instructor. They will be submitted for Sanger sequencing. Next time, we will evaluate the results.

© Katherine Petrie, 2020 | Do not copy or distribute without permission

Lab Session 6. Analyze CRISPR sequences (~ 3 hours)

Here, you'll take a look at the sequences for your CRISPR amplicons. Sanger sequencing can typically only read about 1000 bases, so if you have very long CRISPR loci (recall the size of your PCR product) you may have not have sequence data for all of the CRISPRs. However, a cool property of CRISPR arrays is that new spacer sequences are not randomly inserted, they are always added at the forward (5' end) of the loci. This means that the first spacers you see in your sequence data the most recent ones that were added.

Once you have the sequences, you will run them through a web-based algorithm that will identify the repeat and spacer regions. The unique spacers correspond to the part of the guide RNA that are derived from viral invaders.

Materials

sequence for you and your partner to analyze
computer web browser

Protocol

1. Download your sequence and convert the .seq file into a .fasta file as directed by your instructor.

2. Go to the CRISPRCasFinder website: https://crisprcas.i2bc.paris-saclay.fr/CrisprCasFinder/Index

3. Upload the fasta file you created in to the CRISPRCasFinder website. Leave all settings as default, and click 'Run CRISPRCasFinder' at the bottom.

8. When the results pop up, click the "Details" button. Record the spacer count, and copy and paste the repeat sequence, and the sequence of every spacer into the shared class database as directed by your instructor.

9. One at a time, Use BLAST to find the top **virus** match for each spacer. Be careful, as many of the matches may just be related bacterial strains. Go to https://blast.ncbi.nlm.nih.gov/Blast.cgi and click on "Nucleotide Blast." Paste in a spacer sequence, and click the BLAST button. If you want, you can do this in a higher throughput fashion by pasting a list of spacer sequences, which you can get by clicking the 'display spacers (fasta)' button on the CRISPRCasFinder details page.

- Record the top virus match, and its E-value.

- If you do not get a virus with a significant result (E-value < 0.1), try again (go back to the BLAST page) but this time type viruses under organism before you click blast.

© Katherine Petrie, 2020 | Do not copy or distribute without permission

- If you still do not get a result, record 'no viral matches' in the class database, and make a note of whether or not there are bacterial matches. No viral matches typically means that the virus from which this spacer is derived is new - it's never been sequenced or put into the NCBI database.

10. Once you have added all your spacers to the data sheet and identified them, spend some time researching the following key questions. *Are the CRISPR loci of commercial and heirloom bacteria different? Do they differ in size (according to the PCR amplicons)? Are they different in the types of viruses they are resistant to? Do the results have implications for human health?* Think about both general implications from class data as a whole as well at least one specific implication of one of the particular viruses your strains are immune to. You can use google, google scholar and NCBI to search for more information on the viruses you found. Are they commonly found in the human gut? Where is their host normally found? You can also look at the bacterial matches that came up in your search to learn more about the other types of bacteria that are resistant to the virus to see how widespread resistance is.

© Katherine Petrie, 2020 | Do not copy or distribute without permission
