# Supplementary material for: There’re CRISPRs in My Yogurt: A Discovery-Based CURE at the Intersection of Industrial Food Production and the Human Microbiome
Source: Front Microbiol. 2020 Oct 22;11:578737. doi: 10.3389/fmicb.2020.578737 (PMC7642981; doi:10.3389/fmicb.2020.578737)
Supplement: Supplementary Data Sheet 4 — Lab report rubric. [file Data_Sheet_4.PDF]

Yogurt Module Lab Report - Specific Guidelines. (66 points, 10% of grade)
For this lab report, you should cover what you did **as a group**, not just you and your partner. So it's OK if you share photos, tables, and figures
The page limit is 7 pages, INCLUDING citations, figures, tables and references (it's ok to shrink table font size).

category	0-2 points	3-4 points	5-6 points	7-8 points
Title & Abstract	- abstract is missing more than 1 of the 4 components	- abstract is missing 1 of the 3 components (a-c)	- abstract has a few scientific errors/ misconceptions	- there is a descriptive title (1 pt)<br>- abstract accurately and clearly describes:<br><br>a. what was done<br>b. what the findings were<br>c. why this research question was important

category	0-2 points	3-4 points	5-6 points	7-8 points	9-10 points
Intro-duction	- introduction is missing more than 3 of the components (a-e)	-introduction is missing 3 of components (a-e)	- introduction is missing 2 of components (a-e)	- introduction has a few scientific errors /misconceptions<br>*or*<br>- results are missing 1 of the 5 components (a-e)	Introduction clearly and accurately:<br><br>a. introduces lactic acid bacteria and dairy fermentation<br>b. explains difference between commercial and heirloom yogurt cultures<br>c. introduces CRISPRs as bacterial immune system<br>d. describes 'vaccination' of commercial yogurt strains<br>e. explains why we are comparing commercial vs heirloom strains, including potential significance of results to human health

category	0-2 points	3-4 points	5-6 points	7-8 points	9-10 points
Methods	- 10 or more items needed to reproduce experiments are incorrect or missing	- 6-9 items needed to reproduce experiments are incorrect or missing	- 4-6 items needed to reproduce experiments are incorrect or missing	- 1-3 items needed to reproduce experiments are incorrect or missing	- style is correct (past tense, describes what was done, not step by step recipe) (1 pt)<br>- has enough information for microbiologist to reproduce experiments (include which heirloom yogurt, which brand of commercial yogurt, and which strains are in your commercial yogurt. Do not describe the production of "lab strain" yogurt).
category	0-2 points	3-4 points	5-6 points	7-8 points	9-10 points
Results (this section should have written text that walks the reader through the results, not just a collection of figures and tables).	- results are missing more than 5 of the 6 components (a-f)	- results are missing 4-5 of the 6 components (a-f)	- results are missing 2-3 of the 6 components (a-f)	- results have a few scientific errors/ misconceptions<br>*or*<br>- results are missing 1 of the 6 components (a-f)	- results section accurately and clearly:<br>a. describes 16S sequencing results for one commercial and one heirloom sample.<br>b. describes overall strain screening strategy and results, including cell appearance, gram stain, fermentation and thermotolerance tests<br>c. shows photo of PCR gels & list size (range and estimate) of any successful bands<br>d. list all spacer sequences for the strains your group analyzed without duplicating overlaps<br>e. list the top virus BLAST match for each spacer (write 'no significant match' if that's the case)<br>f. show all PCR band size estimates for our class, use them to compute average and standard deviation for commercial and heirloom, and perform t-test to see if there is a significant difference between commercial and heirloom

category	0-2 points	3-4 points	5-6 points	7-8 points
Discussion	- discussion is missing more than 2 of components (a-e)	- discussion is missing 2 of components (a-e)	- discussion has a few scientific errors/ misconceptions *or*<br>- discussion is missing 1 of components (a-e)	- discussion section accurately and clearly:<br><br>a. interprets 16S data in terms of how heirloom different from commercial and any unexpexted results<br>b. interprets meaning of class-wide results for difference in the CRISPR array size in commercial vs heirloom<br>c. speculates on whether results do or do not have implications for human health (discuss general implications from class data as a whole AND at least one specific implication of one of the particular viruses your strains are immune to)<br>d. discusses limitations of this project and ways to improve the method<br>e. suggests future experimental or literature or database research directions

category	0-2 points	3-4 points	5-6 points
Citations	- more than one of citations are missing, or are not scientific journal articles	- one of 4 citations is missing, or is not a scientific journal article	- has all 4 required citations (must be scientific journal articles)
category	0-2 points	3-4 points	5-6 points
Writing Clarity & Length	- lab report goes OVER the length limit (7 pages, including reference, tables, figures, and photos, single - spaced 12 point font for body)	- there are several places where meaning is not clear	- grammar, spelling, and style do not interfere with understanding
category	0-2 points	3-4 points	
Figures & Tables	- missing 1 or more of the components (a-d).	a. all figures and tables are labeled with a number and title<br>b. all figures and tables referenced in the text<br>c. all figures are good representations of the data, with easy to interpret labels<br>d. Microscope photos are in focus and have a scale bar	

category	0-2 points
Profession alism	- your lab bench was always found in proper order after you left<br>- you were an active participant in your group's lab work and followed all safety rules
category	0-2 points
Scientific Writing	- full genus and species written out first time used<br>- genus and species always italicized<br>- genus always/species never capitalized
